# Choledochoduodenal fistula presenting with pneumobilia in a patient with gallbladder cancer: a case report

**DOI:** 10.1186/1752-1947-6-61

**Published:** 2012-02-14

**Authors:** Elham Dadzan, Hossein Akhondi

**Affiliations:** 1 Transplant Institute, Georgetown University Hospital, Washington, DC, USA; 2 Hospitalist Service, Washington Hospital Center, Washington, DC, USA

## Abstract

**Introduction:**

Spontaneous biliary tract fistulas are rare entities. Most of them are associated with long-standing gallstones (especially common bile duct stones, or recurrent biliary tract infections), some with more uncommon diseases such as gallbladder cancer. Some authors believe that back flow from fistulas predisposes patients to gallbladder cancer and some believe that cancer causes necrosis and fistula formation. Gallbladder cancer has a dismal prognosis and 85% of patients are dead within a year of diagnosis. A common complication of gallbladder cancer is obstruction of the common bile duct, which may produce multiple intra-hepatic abscesses in or near the tumor-laden gallbladder. Fistula formation may further complicate the clinical picture.

**Case presentation:**

We present a case of choledochoduodenal fistula in a 60-year-old diabetic African-American woman with gallbladder cancer. The initial clinical presentation was confusing and complex. Our patient was also found to have a gallbladder fossa abscess that was drained percutaneously as another complicating factor relating to her cancer. She developed myocardial infarction, massive upper gastrointestinal bleeding and respiratory arrest during her stay in hospital. Computed tomography was very helpful in assessing our patient and we discuss how, in a patient with pneumobilia, it can be helpful for detecting fistula, air in bile ducts or to show contractions of the gallbladder.

**Conclusions:**

We believe this case merits reporting as it shows an entity that is not frequently thought of, is hard to diagnose and can be fatal, as in our patient. Careful evaluation, and computed tomography studies and endoscopic retrograde cholangio-pancreatography are helpful in early diagnosis and finding better management options for these patients.

## Introduction

Spontaneous biliary tract fistulas are rare entities, hard to diagnose and difficult to manage. Their differential diagnosis is broad and clinical suspicion is needed for accurate recognition. We present a case of a choledochoduodenal fistula in a woman with gallbladder cancer, and provide a discussion of her diagnosis and management.

## Case presentation

A 60-year-old African-American woman with a medical history significant for diabetes mellitus type 2, hypertension and hypercholesterolemia was evaluated in our hospital for a one-month history of periodic nausea and vomiting. Our patient reported episodes of emesis daily, consisting of ingested food without blood. She denied any abdominal pain, diarrhea, fever or chills, although on the day of admission some epigastric pain and discomfort were present. Her symptoms were progressively worsening.

The results of a physical examination were unremarkable. She was found to have a hypochromic microcytic anemia (hemoglobin 9.3 mg/dL) and leukocytosis at 15,900 cells/μL. Her chloride level was decreased at 74 mEq/L and bicarbonate level was elevated at 37 mEq/L. She also had azotemia with blood urea nitrogen of 88 mg/dL and creatinine of 3.7 mg/dL, all of which were new findings. The results of liver function tests, coagulation studies, and amylase and lipase tests were all within normal ranges.

**Figure 1:**
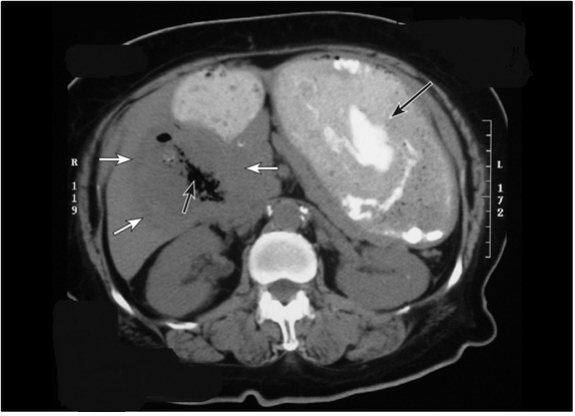
**Computed tomography (CT) scan of the abdomen showing distended stomach compatible with gastric outlet obstruction (long black arrow), gallbladder fossa abscess (area of different density inside the liver) (white arrows), and free air inside the fossa (short black arrow)**.

Ultrasound (US) and computed tomography (CT) of the abdomen showed marked gastric distention and dilatation suggestive of gastric outlet obstruction, air in the biliary tree and an abscess in the gallbladder fossa area (Figure 1). The findings were inconclusive and the assumption at that point was a diagnosis of emphysematous cholecystitis with possible perforation of the gallbladder. A percutaneous drain was placed and it collected approximately 300 cm3 of brownish-black fluid. A fluid smear showed moderate yeast and many Gram-positive diphtheroid rods. Culture showed multiple organisms including: Saccharomyces cerevisiae, Torulopsis *glabrata*, α-hemolytic *Streptococcus *(not *Enterococcus*), and *Clostridium perfringens*. Blood culture results were negative but a urine culture grew yeast (not *Candida*) more than 100,000 cfu/cm^3^. Our patient was started on ampicillin, metronidazole, and fluconazole. Intravenous fluids were also initiated and the pre-renal azotemia improved and her creatinine level returned to 1.7 mg/dL.

After drain insertion, her aspartate transaminase (AST) became elevated to 393 IU/L and alanine transaminase (ALT) to 245 IU/L. A day later, our patient complained of chest and left arm pain as well as increased shortness of breath. An electrocardiogram showed ST segment elevation in the anterior leads consistent with myocardial infarction, and she was noted to be in pulmonary edema. Our Cardiology Department was consulted, and cardiac catheterization showed three vessel disease with left ventricular dysfunction. Balloon dilatation and stenting of the involved vessels was undertaken, with resolution of the symptoms.

**Figure 2:**
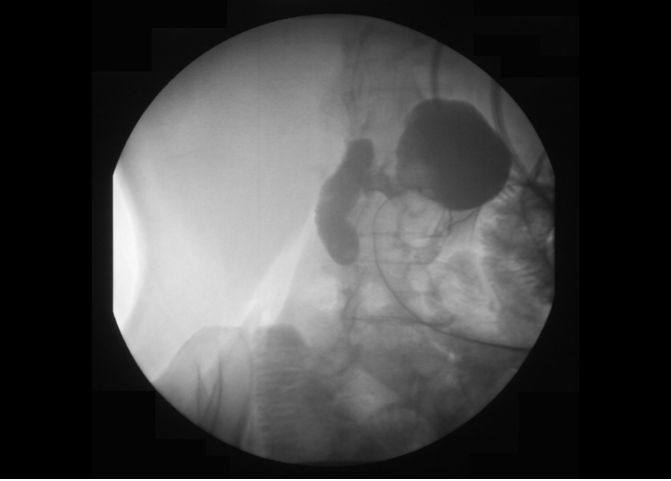
**Barium study of the gastrointestinal tract showing a fistula between the gallbladder and duodenum**.

After this incident, our patient underwent an upper gastrointestinal X-ray study with contrast, which suggested a gallbladder-duodenal fistula (Figure 2). A second drain was then placed to remove the rest of the fluid present in the presumed abscess area.

Our patient then experienced massive upper gastrointestinal bleeding necessitating immediate surgical intervention. An exploratory laparotomy was performed, which showed a tumor involving the gallbladder with invasion of adjacent structures, acute and chronic cholecystitis with cholelithiasis and choledochoduodenal fistula. The tumor was later confirmed to be an adenocarcinoma. Because of the extent of the disease, a partial cholecystectomy was performed with drainage of the duodenal stump, lysis of adhesions and loop gastrojejunostomy.

Our patient developed respiratory failure following surgery, was intubated, and placed on ventilatory support. After several days on mechanical ventilation, she was taken off ventilation at her family's request, and died.

## Discussion

Biliary tract fistulas are rare entities categorized into spontaneous and post-operative types. Spontaneous biliary-enteric fistulas are produced by gallstones (90%), peptic ulcer disease (6%) and malignancy or trauma (4%). The most common communication is cholecystoduodenal (61% to 77%), followed by cholecystocolonic (14% to 17%) and cholecystogastric (6%) [1].

A long-standing history of biliary stones (84 months average), recurrent biliary tract infections (93.8% of cases), and the presence of common bile duct (CBD) stones (88.9%) are factors relevant to the formation of a choledochoduodenal fistula (CDF) when seen with cholelithiasis [2]. Previous biliary surgery is a lesser contributing factor.

CDFs are classified into distal (peripapillar) and proximal types. A distal CDF connects the duodenum to the region within 2 cm of the distal CBD. A proximal CDF drains elsewhere of the biliary system (2 cm and above the junction of the CBD to the papilla). The distal type is far more common and has several cardinal features: its length is less than 1.5 cm, its orifice is around or on the papillary fold, there is prominent pneumobilia, there is less jaundice and larger CBD stones. Presumably, the large stone and large orifice cause the passing of bilirubin and decrease jaundice, but also cause air to enter the biliary system [2]. Proximal CDFs are single in number but distal ones can be multiple.

Patients with CDF lose the barrier of papilla so there is exposure of the biliary system to gut flora and also chronic fluid and electrolyte wasting in the biliary system and malabsorption.

This clinical entity mostly has unusual and deceiving presentations that mimic the symptoms of cholelithiasis. Clinical judgment and expertise is necessary for their discovery, which many times happen unexpectedly during surgery or endoscopic retrograde cholangiopancreatography (ERCP).

Plain films of the abdomen may show air in the biliary tree, which can be seen in 30% of cases of biliary enteric fistula, but is not diagnostic of this entity. Contrast gastrointestinal studies may demonstrate the fistula or reflux of contrast media into the biliary tree during a barium study of the bowel, which is very specific. A finding of an ectopic radiopaque stone that varies in location is also diagnostic, but this is rare (3% of patients). Recently, an indirect sign suggesting fistula was described during ERCP, where biliary tree dilatation subsides when the patient is placed in an anti-Trendelenburg position [3].

CT is a valuable diagnostic method that helps by showing fistulas, air in bile ducts and contraction of the gallbladder. CT imaging of a biliary enteric fistula appears useful for differentiating between a gallbladder-enteric fistula (GB-EF) and a common bile duct-enteric fistula (CBD-EF) [4]. In 13 patients with pneumobilia who had not had surgical biliary-enteric anastomosis or endoscopic sphincterotomy, the presence of the fistula, location of air in the biliary tree and the appearance of the gallbladder were assessed (Table 1). It appeared that differentiation is possible using these parameters.

**Table 1:** Computed tomography (CT) scan results in 13 patients with pneumobilia

Finding	Gallbladder-enteric fistula (seven patients)	Common bile duct-enteric fistula (three patients)	Emphysematous cholecystitis (one patient)	Gallbladder cancer (one patient)	Incompetent sphincter of Oddi (one patient)
Fistula detected	3 (43%)	0 (0%)			
Air detected in the common bile duct	4 (57%)	3 (100%)		1 (100%)	
Contraction of gallbladder	6 (86%)	1 (33%)			

Treatment of a proximal CDF is surgical. For distal cases, endoscopic therapy, with use of a stent or fibrin sealant, is an alternative choice to surgery. The endoscopic features of various CDFs may offer clinical guide for treatment [2]. Gallstone ileus is an unusual complication of a cholecysto-enteric fistula, and is a mechanical bowel obstruction caused by a gallstone impacted in the intestinal lumen. The stone almost always measures 2.5 cm in diameter or more.

Biliary fistulas are rare but they do occur, especially in patients with gallbladder cancer [5-7]; there is a theory that constant back flow from the fistula causes chemical irritation and eventually cancer in the gallbladder.

## Conclusions

Spontaneous biliary fistulas have been associated with gallbladder cancer; if they are the cause of cancer, or a complication of it, this has not yet been defined. Presentations are different and deceiving, and good clinical judgment is needed for diagnosis and management. CT and ERCP have a role in assessing these patients.

## Consent

Written informed consent was obtained from the patient's next of kin for publication of this case report and any accompanying images. A copy of the written consent is available for review by the Editor-in-Chief of this journal.

## Competing interests

The authors declare that they have no competing interests.

## Authors' contributions

HA encountered the case and compiled this report. ED prepared and edited the discussion and abstract. Both authors read and approved the final manuscript.
